# Supplementary material for: Open-top multisample dual-view light-sheet microscope for live imaging of large multicellular systems
Source: Nat Methods. 2024 Mar 20;21(5):798–803. doi: 10.1038/s41592-024-02213-w (PMC11093739; doi:10.1038/s41592-024-02213-w)
Supplement: Supplementary file 1 — 1. Assessment of image quality and dual-view fusion, 2. Perturbation experiment, 3. Methods, 4. Tables, 5. Figures and 6. References. [file 41592_2024_2213_MOESM1_ESM.pdf]

nature methods

Brief Communication https://doi.org/10.1038/s41592-024-02213-w

Open-top multisample dual-view light-sheet microscope for live imaging of large multicellular systems

In the format provided by the authors and unedited

1. Assessment of image quality and dual view fusion

To assess the improvement in image quality by using two opposing detection objectives, we compared the image quality with increasing imaging depth by calculating the Shannon Entropy of the Discrete Cosine Transform (DCT) [1] for each z-section of both detection objectives individually and the fused data. As example we performed this comparison with an intestinal and human colon cancer organoid (Extended Data Fig 5a-d). Both model systems show a clear decrease in image quality with increasing imaging depth and distance from the detection objective (Extend ended Data Fig 5b,d), which can be compensated by combining the data from two opposing objectives. Additionally, we compared the image quality of our developed system with another open-top light sheet microscope published in [2]. This system offers dual illumination (Nikon 10X, NA 0.3) and one detection objective (Nikon 25X, NA 1.1). We imaged the same gastruloid stained with the nuclear marker DRAQ5 in both systems (Extended Data Fig 5e, g) and evaluated the image quality by calculating Shannon Entropy of the DCT. The single-detection system shows a degrading image quality score with increasing imaging depth (Extended Data Fig 5f), whereas our microscope enables a higher image quality throughout the sample due to the opposing detection objectives (Extended Data Fig 5h). Together, these results show the importance of dual detection to guarantee high image quality in large specimens.

To demonstrate the spatial overlap of the two views, we imaged gastruloids stained for DRAQ5 and fused the two views without a sigmoidal function. There is no visible misalignment on the fused image near the switching point between views (Supplementary Fig 1).

2. Perturbation experiment

Our sample mounting strategy also enables experiments with parallel perturbations. During the same imaging experiment, each of the four chambers can be used for a particular condition with many individual positions imaged within each chamber. We assessed the mechano-osmotic effects of prostaglandin E2 (PGE) [3], [4], Forskolin (a CFTR activator) [5], and a hyperosmotic shock induced by NaCl on intestinal organoids. The high temporal resolution of 3 minutes (Supplementary Video 10) revealed rapid organoid inflation and contraction in response to the respective treatments.

3. Supplementary Methods

Human material, mouse and stem cell lines:
Human organoids:
Patient-derived organoids identified by the HUB code P-19bT CRC organoids are cataloged at www.huborganoids.nl and can be requested at techtransfer@huborganoids.nl. Distribution to third (academic or commercial) parties has to be authorized by the Biobank Research Ethics Committee of the University Medical Center Utrecht (TCBio) at request of Hubrecht Organoid Technology (HUB). The movieSTAR, is based on a transposase-based integration method (movieSTAR: Tol2 insulator8xSTAR-min.pLGR5-sTomato-NLS-pA-PGK-H2BmNeonGreen-2A-Puro) as previously described [6].

Mouse Strains:
All animal experiments were approved by the Basel Cantonal Veterinary Authorities and conducted in accordance with the Guide for Care and Use of Laboratory Animals. Male and female outbred mice between 7 and 15 weeks old were used for all experiments. The study employed the following mouse lines: B6/N x R26 Fucci2 (Tg/+) intestines, generously provided by J. Skotheim at Stanford University, to generate organoids. These organoids were subsequently infected with pGK Dest H2B-miRFP670 (Catalog no. 90237, Addgene). Additionally, (C57BL/6J) WT mice, R26-mG mice (#007676 [7]), obtained as a kind gift from T. Hiiragi at the Hubrecht Institute, R26-H2B-mCherry mice [8], received as a kind gift from C. Tsiairis at FMI Basel, and R26-mG/H2B-mCherry (heterozygous) mice, generated in-house following the procedure described in reference [9], were utilized. To monitor cell and nuclear shape, homozygous R26-mG mice were crossbred with homozygous R26-H2B-mCherry mice.

For genotyping of the newly generated R26-mG/H2B-mCherry mouse strain, the One Taq HS QuickLoad PCR reagent was used (Catalog no. M0488L, NEB). The genotyping process utilized the primer sets and cycling conditions listed in Supplementary Table 3.

Mouse stem cell lines:
Cloning:
As a nuclear marker H2B-miRFP was used. The protein coding sequence of H2B was amplified from the pCAG-H2BtdiRFP-IP plasmid obtained from Addgene (Catalog no.47884, Addgene) using KAPA HiFi polymerase (Catalog no. KK2501, Kapa Biosystems) introducing restriction sites for XhoI (Catalog no. R0146s, NEB) and SmaI (Catalog no. R0141S, NEB). These sites were subsequently used to introduce the PCR product into linearized pmiRFP670-N1 plasmid (Catalog no.79987, Addgene) using T4 DNA Ligase (Catalog no. M0202S, NEB). The protein coding sequence of H2B-miRFP670 was then amplified via PCR introducing attB sites to eventually clone the PCR product into pDONR221 (Catalog no. 12536017, Thermo Fisher) using Gateway BP Clonase II (Catalog no. 11789020, Thermo Fisher) creating an entry clone. Using LR Clonase II (Catalog no. 11791100, Thermo Fisher) the sequence of interest was introduced into an expression clone making use of pPB-UbC-DEST-pA-pgk-hph (kind gift from J. Betschinger, Novartis) creating the final PiggyBac construct containing H2B-miRFP670 driven by an Ubc promoter and a hygromycin selection cassette (hph) under a pgk promoter flanked by 5` and 3` repeats to allow PBase insertion.

As a membrane marker a Lck-tagged GFP was used. First, a pPB-UbC-DEST-pA-pgk-hph vector (kind gift from J. Betschinger, Novartis) based on plasmids published in [10] was subcloned to introduce a single MluI cloning site. To do so, the initial backbone was linearized with XbaI (Catalog no. R0145T, NEB) and BbsI (Catalog no. R0539S, NEB) and a gBlock (IDT) was used to introduce the MluI cut site via Gibson assembly (Catalog no. E2611L, NEB). The protein coding sequence of Lck-GFP was amplified from the Lck-GFP plasmid (Catalog no.61099, Addgene) using Phusion high fidelity polymerase (Catalog no. F530S, Thermo Fisher). Using MluI (Catalog no. R3198S, NEB) the expression vector was

linearized, and Gibson assembly was used to incorporate the LcK-GFP sequence into the final PiggyBac vector.

The Qiaquick gel extraction kit (Catalog no. 28706, Qiagen) was used to purify PCR and digestion products from agarose gels. Mix & Go E. Coli transformation kit (Catalog no. T3001, Zymo Research) treated DH5alpha cells (Catalog no. 18265-017, Invitrogen) were used for transformation and plasmid amplification. Bacterial selection was performed with Kanamycin (50 µg/ml) for Addgene plasmids #61099 and #79987 and Ampicillin (100 µ/ml) for all other plasmids. Plasmid purification was performed using QIAprep Spin Miniprep Kit (Catalog no. 27106, Qiagen) and NucleoBond Xtra Midi kit (Catalog no. 740410.50, Macherey-Nagel).

Engineering of monoclonal mouse embryonic stem cell lines:
Embryonic stem cell lines E14 and CGR8 (see below) were transfected using Lipofectamine2000 (Catalog no. 11668030, Thermo Fisher Scientific) to deliver PiggyBac plasmids. A total of 800 ng of purified DNA (400ng of expression vector and 400 ng of the vector carrying the PiggyBac transposase (kind gift form J. Betschinger, Novartis)) were diluted in 50 µl of OptiMEM (Catalog no. 31985062, Gibco)). In a separate tube 2 µl of Lipofectamine2000 was diluted in 50 µl. After 5 min of incubation at room temperature both mixtures were mixed before subsequent incubation at room temperature for 20 min. In the meantime, $2.5x10^5$ cells were seeded into a gelatine coated well of a 24well plate containing Serum medium. Serum medium consists of GMEM (Catalog no.G5154, Sigma) supplemented with 10% v/v ESC-grade FBS (Catalog no. 16141079, Invitogen), 1x GlutaMAX (Catalog no. 35050038, Invitrogen), 1x MEM-NEAA (Catalog no. M7145-100ML, Sigma), 1x sodium pyruvate (Catalog no. 11360070, Invitrogen), 1x β-Mercaptoethanol (Catalog no. 21-985-023, Fisher Scientific), 3 µM CHIR99021 (Chir) (Catalog no. 72054, Stem Cell Technologies), 1 µM PD0305901 (Catalog no. 100-0248, Stem Cell Technologies) and 0.01 µg/ml LIF (Catalog no. 78056, Stem Cell Technologies). While the cells were still in suspension, the transfection mix was added and the plate was agitated in order to equally distribute the solution. Now the plate was incubated over night before the medium was replaced with fresh Serum medium. After 3 days, antibiotic selection with hygromycin B (Catalog no. 10-687-010, Fisher Scientific) (200 µg/ml) was started and continued for >7 days. Finally single cells were sorted into a gelatin coated 96well plate containing Serum medium supplemented with Pen/Strep (Catalog no. 15140122, Gibco). Based on fluorescence and morphology, monoclonal colonies were selected and expanded.
Embryonic stem cell lines CGR8-Lck-GFP-H2B-mCherry and E14-H2B-mCherry were generated from their parental lines CGR8 and E14 respectively. Both E14 and CGR8 cell lines are of 129P background and were kind gifts from M. Lutolf (IHB Roche). Cells were tested routinely for mycoplasma via PCR.

Sample preparation
Parotid salivary gland organoids:
Parotid salivary glands were dissected following previously established protocols [11]. After dissection, the gland tissue was cut into small fragments using a razor blade. Thereafter, fragments were enzymatically digested with Trypsin 0.05% EDTA for 10-15 min. Cells were passed through a 40um strainer, and embedded in 50% Matrigel (for organoid culture, Catalog no. 356255, Corning) and supplied with Basal culture medium consisting of DMEM/F12 (with 15 mM HEPES) (Catalog no. 36254, Stem Cell Technologies), 1x GlutaMAX (Catalog no. 35050038, Thermo Fisher Scientific)), and 100 µg/mL Pen/Strep (Catalog no. 15140-122, Gibco)) with 0.5 nM Wnt (NGS) (Catalog no. N001, UproteinExpress), 1 µg/ml Recombinant R-Spondin1 (kind gift from Novartis), 100 ng/ml Recombinant Noggin (Catalog no. 250-38, PrepoTech), 1x B27 supplement (Catalog no. 17504044, Thermo Fisher Scientific), 1.25 mM NAC (Catalog no. A7250-100G, Sigma Aldrich), 50 ng/ml hEGF (Catalog no. 236-EG-200, RnD Systems), 10 ng/ml hNRG1 (Catalog no.5898-NR-050, RnD Systems), 0.5 µM A83-01 (Catalog no. 2939, Tocris), 100 ng/ml hIGF1 (Catalog no.291-G1-200, RnD Systems), 50 ng/ml hFGF2

(Catalog no. 233-FB-025, RnD Systems), and 10 µM ROCK-Inhibitor (Catalog no. Y-27632, Stem Cell Technologies). Organoids were maintained by hard splitting every week. Directly after the initial hard-split the culture medium composition changed to 500 ng/ml Recombinant R-Spondin1, 100 ng/ml Recombinant Noggin, 1x B27 supplement, 1.25 mM NAC, 1 ng/ml hNRG1, 100 ng/ml hIGF1, 50 ng/ml hFGF2, and 100 µg/ml Primocin (Catalog no. ant-pm-1, Invivogen). Samples on the light-sheet were imaged 7 days after a hard-split.

Human colon cancer organoids:
Human CRC organoids were maintained in 70% Matrigel-drops (for organoid culture, Catalog no. 356255, Corning). The medium composition for the patient-derived tumor organoid was: Basal culture medium (see parotid salivary gland organoid section) supplemented with 0.5 µg/mL recombinant R-Spondin1 (kind gift from Novartis), 100 ng/mL recombinant Noggin (Catalog no. 250-38, PrepoTech), 1x B27 supplement (Catalog no. 17504044, Thermo Fischer Scientific), 1.25 mM NAC (Catalog no. A7250-100G, Sigma Aldrich), 50 ng/mL hEGF (Catalog no. 236-EG-200, RnD Systems), 100 ng/mL hIGF1 (Catalog no. 291-G1-200, RnD Systems), 50 ng/mL hFGF2 (Catalog no. 233-FB-025, RnD Systems), 10 nM Gastrin (Catalog no. G9145-.1MG, Sigma Aldrich), 500 nM A83-01 (Catalog no. 2939, Tocris), 3 uM SB202190 (Catalog no. 1264, Tocris), and 100 µg/ml Primocin (Catalog no. ant-pm-1, Invivogen). Preceding imaging using the light-sheet microscope setup, organoids were dissociated into individual cells and then embedded into 60% Matrigel drops within a specialized holder designed for light-sheet imaging experiments. The culture medium, mentioned earlier, was supplemented with 10 µM ROCK-Inhibitor (Catalog no. Y-27632, Stem Cell Technologies) during the first 4-days. Subsequently, medium renewal occurred every 48 hours without the ROCK-Inhibitor.

Hepatic organoids:
The establishment of murine hepatic organoid culture was carried out with some adaptations as described in [12]. In brief, a hepatectomy was performed on a mouse from the above described R26-mG/H2B-mCherry strain (see mouse strain section above). After thoroughly washing the explanted liver in ice cold PBS the tissue was minced using surgical scissors. After a fine tissue paste formed, the material was resuspended in 50 ml of ice cold DMEM (high glucose, Catalog no. D5796-500ML, Sigma Aldrich) and transferred into a 50 ml tube. After inverting the tube multiple times, the material was sedimented via centrifugation (80 g at 4°C for 2 min). This step was repeated 3 times. Now the supernatant was decanted, with around 10 ml remaining. The tissue fragments were then further dissociated by extensive pipetting with a 5 ml serological pipette (pipetted up and down at least 30 times). Centrifugation (200 g at 4°C for 5 min) was used to pellet the dissociated tissue before the pellet was resuspended in 15 ml prewarmed digestion mixture (DMEM high glucose supplemented with collagenase (Catalog no. C9407-25MG, Sigma Aldrich) at a final concentration of 0.25 mg/ml. After one more round of centrifugation the pellet was resuspended in 45 ml of prewarmed digestion mixture and incubated at 37°C with moderate shaking for 45 min. After 45 min an aliquot of the suspension was assessed via microscopy to confirm the presence of ductal structures. Now the digestion was terminated by pelleting the supernatant (centrifugation at 4°C and 200 g for 5 min). The pellet was subsequently resuspended in wash medium and centrifuged again with the same settings. Finally, the material was resuspended in 50 % Matrigel (for organoid culture, Catalog no. 356255, Corning) and Hepaticult (Catalog no. 06030, Stem cell technologies) and plated in 50 µl droplets in a 12well tissue culture plate. After the Matrigel drops were solidified 1 ml of Hepaticult medium was added to each well. Medium was changed when passaging the organoids. Organoids were passaged at least 5 times before starting light sheet acquisitions with the newly established organoid line. For light sheet sample mounting 10 µl droplets of mechanically split organoids resuspended in 50 % Matrigel were seeded into a sample holder with a diameter of 1 mm. After solidifying for 15 min at 37°C the Matrigel droplets were covered with 300 µl of Hepaticult.

Intestinal organoids:
Mouse small intestinal organoids were established and cultured as previously described in [2].
Intestinal organoids expressing the Fucci2-reporter (see mouse strain section above) were previously established as described in [2], [13], cultured in droplets of 50% Matrigel (for organoid culture, Catalog no. 356255, Corning) with IntestiCult Organoid Growth Medium (Catalog no. 06005, Stem cell technologies) and were kept in IntestiCult OGM supplemented with 100 µg/ml Pen/Strep (Catalog no. 15140122, Gibco) for maintenance. For light sheet experiments, organoids were collected 5 days after mechanical disruption. Organoids were embedded in Matrigel and ENR medium (1:1 ratio) in custom FEP-chambers. ENR medium is composed of advanced DMEM/F-12 with 15mM HEPES (Catalog no. 36254, Stem Cell Technologies) supplemented with 100 µg/ml Pen/Strep (Catalog no. 15140-122, Gibco), 1× Glutamax (Catalog no. 35050061, Gibco), 1× B27 (Catalog no. 17504044, Gibco), 1x N2 (Catalog no. 17502048, Thermo Fisher Scientific), 1 mM NAC (Catalog no. A7250-100G, Sigma Aldrich), 500 ng/ml R-Spondin (kind gift from Novartis), 100 ng/ml Noggin (Catalog no. 250-38, PrepoTech) and 100 ng/ml murine EGF (Catalog no. 315-09-100ug, RnD Systems). After 20 min of solidification at 37°C, 300 µl of ENR medium supplemented with 20 % Wnt3a-conditioned medium (Wnt3a-CM) was added. Perturbation studies on mouse small intestinal organoids were performed in ENR medium supplemented with 0.5 µM PGE, 5 µM Forskolin, 250 mM NaCl solution, or DMSO as vehicle control.

Hydra:
Light sheet imaging of regenerating *Hydra* was performed on the *ecto[β-act::RFP]/endo[β-act::GFP]* "*Reverse Watermelon"* line of *Hydra vulgaris* [14]. *Hydra* culture was maintained at 18°C in Volvic mineral water. Animals were fed three times per week with freshly hatched *Artemia nauplii*.
For the light sheet acquisition, adult, non-budding animals were used. Animals were transferred to *Hydra* medium (1 mM $CaCl_2$, 0.2 mM $NaHCO_3$, 0.02 mM KCl, 0.02 mM $MgCl_2$, and 0.2 mM Tris-HCl (pH 7.4)) and spheroids were cut as previously described [15]. In short, the animals were bisected with the initial cut being directly under the tentacle ring. Two tissue rings were obtained from each animal by sequentially cutting the body axis. The rings were split in two to three rectangular pieces. The tissue fragments were left to fold for 4 hours in dissociation medium (3.6 mM KCl, 6 mM CaCl2, 1.2 mM MgSO4, 6 mM sodium citrate, 6 mM sodium pyruvate, 4 mM glucose, and 12.5 mM N-tris(hydroxymethyl)methyl-2-aminoethanesulfonic acid (pH 6.9)) at room temperature. Properly closed spheroids of a typical size (300-500 µm in diameter) were selected for the imaging. The *Hydra* were imaged in sample chambers that were filled with 500 µl *Hydra* medium.

Gastruloids:
Gastruloids used in this study have been prepared as previously described [16]. In brief mouse embryonic stem cells (ESC) were maintained on gelatin-coated culture plates (6-well) in N2B27 medium consisting of 50 % DMEM/F12 (Catalog no. 21331020, Gibco) and 50 % Neurobasal medium (Catalog no. 21103049, Gibco) supplemented with 1x N2 (homemade) and 1x B27 serum free supplement (Catalog no. 17504044, Gibco), 1x GlutaMAX (Catalog no. 35050061, Gibco), HEPES (Catalog no. 83264, Sigma Aldrich) and 1x β-mercaptoethanol (Catalog no. 21-985-023, Gibco). For stem cell maintenance N2B27 medium was supplemented with 3 µM CHIR99021 (Chir) (Catalog no. 72054, Stem Cell Technologies), 1 µM PD0305901 (Catalog no. 100-0248, Stem Cell Technologies) and 0.01 µg/ml LIF (Catalog no. 78056, Stem Cell Technologies). ESCs were split every other day by disassociating colonies using 400 µl of Accutase (Catalog no. A6964-500ML, Sigma Aldrich). After visual inspection 2 ml of wash medium (DMEM/F12 with 0.1% BSA Catalog no. 15260037, Thermo Fisher Scientific) was added to create a cell suspension. Cells were pelleted via centrifugation (300 g, 4°C, 5 min) and resuspended in N2B27 medium. This step was repeated one more time to remove remaining traces of compounds added to maintain naïve pluripotency. Cells were usually split in a ratio between 1:10 and 1:15. An aliquot of the same cell suspension was used for cell counting (TC20, Biorad).
40 µl of N2B27 containing 300 ESCs were seeded into each well of a 96well ultra-low attachment U-bottom plate (Catalog no. 7007, Corning). After 48 hours, the formed aggregates were pulsed with

150 µl of N2B27 supplemented with 3 µM Chir for 24 hours. After a total 72 hours post seeding, 140 µl of N2B27 was removed and 160 µl of fresh N2B27 medium was added. At 96 hours post seeding a last medium change was performed removing 150 µl of medium and replacing it with an equal amount of fresh N2B27 medium.
Gastruloid culture was not performed for longer than 120 hours. For light sheet imaging gastruloids have been collected at either 42, 66, 90 or 96 hours post seeding. Gastruloids have been mounted into pockets of custom thermoformed sample holders (Extended Data Fig. 4) holding 500 µl of N2B27 for suspension culture. For indicated cases the pockets were covert with 40 % of Matrigel (for organoid culture, Catalog no. 356255, Corning) diluted in N2B27 to ensure minimal mechanical rotations of forming gastruloids. For the 42 and 66 h timepoints 5 % of fluorescent cells were mixed with 95 % of their respective non fluorescent parental line. For later timepoints 10 % of fluorescent cells were used.

Endpoint immunofluorescence/ DRAQ5staining
Small intestinal organoids:
After live imaging of murine intestinal organoids expressing the Fucci2-reporter (see above), the sample holder was removed from the microscope in order to perform sample fixation using 4 % PFA (Catalog no. 15714, Electron Microscopy Sciences) and 0.08 % glutaraldehyde (Catalog no. 16019, Electron Microscopy Sciences) diluted in PBS for 25 min at room temperature. Next, samples were washed with PBS 3 times with 10 min incubations. In order to reduce auto fluorescent background potentially resulting from the use of glutaraldehyde a quenching step using sodium borohydrate (Catalog no. 452882-5G, Sigma Aldrich) (0.01 g/10 ml of PBS) was performed for 10 minutes at room temperature. Now the samples were permeabilized and blocked by incubating with 3 % donkey serum (Catalog no. D9663-10ML, Sigma Aldrich) and 2 % TritonX (Catalog no. T9284, Sigma Aldrich) in PBS for 1 hour. After permeabilization and blocking primary antibody solution using sheep anti Dll1 (Catalog no. AF3970, RnD Systems) and rabbit anti Lysozyme (Catalog no. A0099, Dako) antibodies at a concentration of 1:100 in PBS supplemented with 3 % donkey serum and 0.1 % TritonX was added. Primary antibody solution was incubated for 2x over night at 4°C before the sample was washed for 3x30 min with PBS at room temperature. Now, the samples were incubated with Fab fragments at a concentration of 1:250 in PBS supplemented with 3 % donkey serum and 0.1 % TritonX overnight. Donkey anti rabbit Fab fragments conjugated to Alexa 647 and donkey anti goat Fab fragments conjugated to Alexa 488 fluorophores were used (Catalog no. 705-607-003 and 711-547-003, Jackson Immuno Research). After washing the samples with PBS for 3x30 min at room temperature, the sample holder was remounted onto the light sheet microscope for imaging.

Gastruloids:
Gastruloids were fixed in 4% PFA (Catalog no. 15714, Electron Microscopy Sciences) in PBS for 30 min at room temperature. After extensive washes with PBS, gastruloids were permeabilized with 1% Triton X (Catalog no. T9284, Sigma Aldrich) in PBS for 1 hour before an overnight incubation with DRAQ5 (Catalog no. 62251, Thermo Fisher Scientific) at a final dilution of 1:300 in PBS. After further extensive washing with PBS, gastuloids were mounted for light sheet imaging.

4. Supplementary Tables

Supplementary Table 1: Characterization of our microscope in comparison to other state of the art systems

Parameter	
**Measured lateral resolution, limited by sampling [µm]**	0.8
**Axial resolution [µm]**	2.9
**Light sheet beam waist [µm]**	3.5
**Pixel spacing [µm]**	0.406
**Field of view [µm] X [µm]**	935 x 935
**Magnification**	16
**Detection NA**	0.8
**Illumination NA**	0.2
**Effective illumination NA**	0.06
**Volumetric Rate**	200 planes in 10 – 30 sec
**Light sheet generation**	Scanned Gaussian beam

Supplementary Table 2: Imaging settings used for all experiments.

Figure	Sample	Fluorescent label	Excitation [nm]	Exposure Time [ms]	Z-Sections [2 um spacing]	Time Interval [mins]	Acquisition time [h]
**1g, 2a-c, Ext Dat. 1a, Supp Video 1**	Intestinal organoids	hGem-mVenus hCdt1-mCherry	515 561	30 10	181 X 2µm	10	67,3
**1 I, Supp. Video 3**	*Hydra*	ecto[[β-act::RFP]	561	10	401 X 2µm	10	67,16
**1 k, Supp Video 5**	Liver organoids	mg-GFP H2B-mCherry	488 561	10 10	201 X 2µm	10	31,3
**1 l, Supp. Video 7**	Human colon cancer organoids	H2B-mNeon STAR-sTom-NLS	488 561	10 10	201 X 2µm	30	137
**2 h,l**	Gastruloids	Lck-GFP	488	10	350 X 1µm	10	5,5
**Ext. Dat. 2b, Supp Video 8**	Parotid salivary gland organoids	H2B-mCherry	561	10	201 X 2µm	30	74,5
**Ext. Dat. 2c, Supp Video 9**	Gastruloids	H2B-iRFP	638	10	251 X 2µm	15	13,5
**Supp. Video 10**	Intestinal organoids	mg-GFP H2B-mCherry	488 561	10 10	121 X 2µm	3	4,9
**Supp. Video 12**	Human colon cancer organoids	H2B-mNeon	561	100	201 X 2µm	30	280,5
**Supp. Video 11**	Intestinal organoids	H2B-mCherry	561	10	181 X 2µm	10	21,5

Supplementary Table 3: Primer sets and cycling conditions used for genotyping the newly generated R26-mG/H2B-mCherry mouse strain

**R26-mG**		**PCR Cycle Conditions**	
**Jackson Laboratory,** [7]		3 min	95°
oIMR7318	CTCTGCTGCCTCCTGGCTTCT	30 sec	95°C
oIMR7319	CGAGGCGGATCACAAGCAATA	30 sec	58°C
oIMR7320	TCAATGGGCGGGGGTCGTT	30 sec	72°C
Expected band sizes:		Cycle no.	34
Knock in allele: 250bp		5 min	72°
WT allele: 330bp			
**R26-H2B-mCherry**		**PCR Cycle Conditions**	
**FMI-mouse facility,** [8]		2 min	95°
R26-P3	TCCCTCGTGATCTGCAACTCCA GTC	30 sec	94°C
R26-P4	AACCCCAGATGACTACCTATCC TCC	30 sec	65°C
R26-P6	GCTGCAGGTCGAGGGACC	30 sec	68°C
Expected band sizes:		Cycle no.	35
Knock in allele: 270bp		5 min	68°
WT allele: 217bp			

5. Supplementary Figures

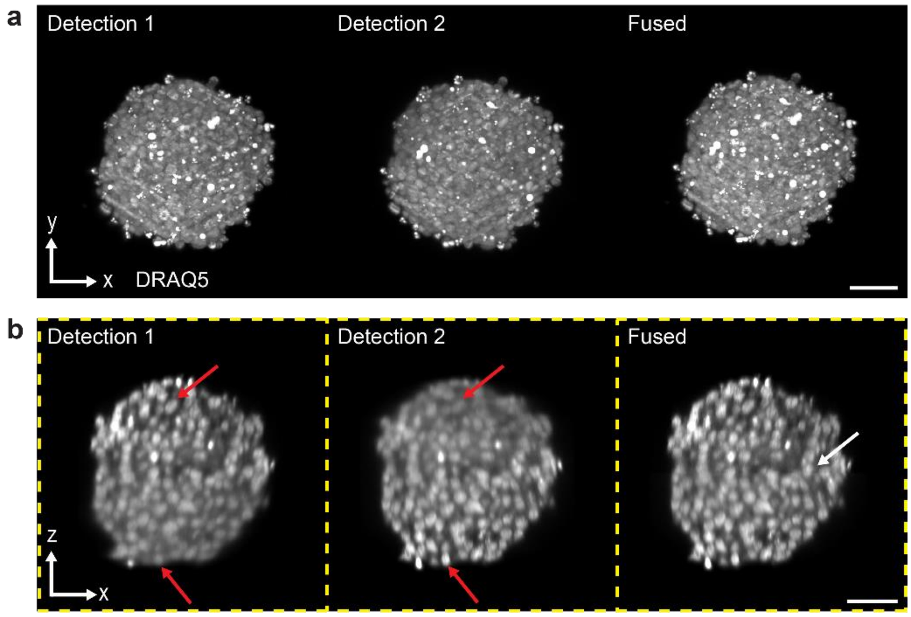

Supplementary Figure 1: Exemplary demonstration of the spatial overlap of the two views

a) MIPs along the Z axis of a gastruloid stained for DRAQ5 using Detection 1, Detection 2 or the fused data. In total n=7 gastruloids were imaged. Scale bar 50 µm.

b) XZ section of the gastruloid shown in e). The data was fused without a sigmoidal function and therefore a discrete switching point is present (white arrow). There is no visible misalignment around the switch point and cells in view 1 and view 2 correspond to each other (red arrow). Scale bar 50 µm.

6. Supplementary References

[1] L. A. Royer *et al.*, “Adaptive light-sheet microscopy for long-term, high-resolution imaging in living organisms,” *Nature Biotechnology*, vol. 34, no. 12, pp. 1267–1278, Dec. 2016, doi: 10.1038/nbt.3708.

[2] D. Serra *et al.*, “Self-organization and symmetry breaking in intestinal organoid development,” *Nature*, vol. 569, no. 7754, pp. 66–72, May 2019, doi: 10.1038/s41586-019-1146-y.

[3] Q. Yang *et al.*, “Cell fate coordinates mechano-osmotic forces in intestinal crypt formation,” *Nature Cell Biology*, vol. 23, no. 7, pp. 733–744, Jul. 2021, doi: 10.1038/s41556-021-00700-2.

[4] E. Ricciotti and G. A. FitzGerald, “Prostaglandins and inflammation.,” *Arterioscler Thromb Vasc Biol*, vol. 31, no. 5, pp. 986–1000, May 2011, doi: 10.1161/ATVBAHA.110.207449.

[5] S. F. Boj *et al.*, “Forskolin-induced Swelling in Intestinal Organoids: An In Vitro Assay for Assessing Drug Response in Cystic Fibrosis Patients.,” no. 120, Feb. 2017, doi: 10.3791/55159.

[6] M. C. Heinz *et al.*, “Liver Colonization by Colorectal Cancer Metastases Requires YAP-Controlled Plasticity at the Micrometastatic Stage.,” *Cancer Res*, vol. 82, no. 10, pp. 1953–1968, May 2022, doi: 10.1158/0008-5472.CAN-21-0933.

[7] M. D. Muzumdar, B. Tasic, K. Miyamichi, L. Li, and L. Luo, “A global double-fluorescent Cre reporter mouse.,” *Genesis*, vol. 45, no. 9, pp. 593–605, Sep. 2007, doi: 10.1002/dvg.20335.

[8] T. Abe *et al.*, “Establishment of conditional reporter mouse lines at ROSA26 locus for live cell imaging.,” *Genesis*, vol. 49, no. 7, pp. 579–590, Jul. 2011, doi: 10.1002/dvg.20753.

[9] R. Niwayama *et al.*, “A Tug-of-War between Cell Shape and Polarity Controls Division Orientation to Ensure Robust Patterning in the Mouse Blastocyst,” *Developmental Cell*, vol. 51, no. 5, pp. 564-574.e6, 2019, doi: https://doi.org/10.1016/j.devcel.2019.10.012.

[10] J. Betschinger, J. Nichols, S. Dietmann, P. D. Corrin, P. J. Paddison, and A. Smith, “Exit from pluripotency is gated by intracellular redistribution of the bHLH transcription factor Tfe3.,” *Cell*, vol. 153, no. 2, pp. 335–347, Apr. 2013, doi: 10.1016/j.cell.2013.03.012.

[11] C. Watermann *et al.*, “Step-by-step protocol to perfuse and dissect the mouse parotid gland and isolation of high-quality RNA from murine and human parotid tissue.,” *Biotechniques*, vol. 60, no. 4, pp. 200–203, Apr. 2016, doi: 10.2144/000114404.

[12] L. Broutier *et al.*, “Culture and establishment of self-renewing human and mouse adult liver and pancreas 3D organoids and their genetic manipulation,” *Nature Protocols*, vol. 11, no. 9, pp. 1724–1743, Sep. 2016, doi: 10.1038/nprot.2016.097.

[13] G. de Medeiros *et al.*, “Multiscale light-sheet organoid imaging framework,” *Nature Communications*, vol. 13, no. 1, p. 4864, Aug. 2022, doi: 10.1038/s41467-022-32465-z.

[14] K. M. Glauber *et al.*, “A small molecule screen identifies a novel compound that induces a homeotic transformation in Hydra.,” *Development*, vol. 140, no. 23, pp. 4788–4796, Dec. 2013, doi: 10.1242/dev.094490.

[15] J. Ferenc and C. D. Tsiairis, “Studying Mechanical Oscillations During Whole-Body Regeneration in Hydra.,” *Methods Mol Biol*, vol. 2450, pp. 619–633, 2022, doi: 10.1007/978-1-0716-2172-1_33.

[16] S. Suppinger *et al.*, “Multimodal characterization of murine gastruloid development,” *Cell Stem Cell*, vol. 30, no. 6, pp. 867-884.e11, Jun. 2023, doi: 10.1016/j.stem.2023.04.018.
