# Supplementary material for: Open-top multisample dual-view light-sheet microscope for live imaging of large multicellular systems
Source: Nat Methods. 2024 Mar 20;21(5):798–803. doi: 10.1038/s41592-024-02213-w (PMC11093739; doi:10.1038/s41592-024-02213-w)
Supplement: Supplementary file 2 — Reporting Summary [file 41592_2024_2213_MOESM2_ESM.pdf]

nature portfolio

Corresponding author(s): Prisca Liberali, Petr Strnad

Last updated by author(s): Jan 18, 2024

Reporting Summary

Nature Portfolio wishes to improve the reproducibility of the work that we publish. This form provides structure for consistency and transparency in reporting. For further information on Nature Portfolio policies, see our Editorial Policies and the Editorial Policy Checklist.

Statistics

For all statistical analyses, confirm that the following items are present in the figure legend, table legend, main text, or Methods section.

n/a	Confirmed	
☐	☒	The exact sample size (*n*) for each experimental group/condition, given as a discrete number and unit of measurement
☐	☒	A statement on whether measurements were taken from distinct samples or whether the same sample was measured repeatedly
☒	☐	The statistical test(s) used AND whether they are one- or two-sided<br>*Only common tests should be described solely by name; describe more complex techniques in the Methods section.*
☒	☐	A description of all covariates tested
☒	☐	A description of any assumptions or corrections, such as tests of normality and adjustment for multiple comparisons
☐	☒	A full description of the statistical parameters including central tendency (e.g. means) or other basic estimates (e.g. regression coefficient) AND variation (e.g. standard deviation) or associated estimates of uncertainty (e.g. confidence intervals)
☒	☐	For null hypothesis testing, the test statistic (e.g. *F*, *t*, *r*) with confidence intervals, effect sizes, degrees of freedom and *P* value noted<br>*Give P values as exact values whenever suitable.*
☒	☐	For Bayesian analysis, information on the choice of priors and Markov chain Monte Carlo settings
☒	☐	For hierarchical and complex designs, identification of the appropriate level for tests and full reporting of outcomes
☒	☐	Estimates of effect sizes (e.g. Cohen's *d*, Pearson's *r*), indicating how they were calculated

Our web collection on statistics for biologists contains articles on many of the points above.

Software and code

Policy information about availability of computer code

Data collection	Microscope control software (Viventis Microscopy), v2.0.0.2
Data analysis	The microscope was designed using Solidworks 2018 SP 1.0.<br>Data analysis and processing was performed with Python 3.10.9 using the following open-source libraries:<br>scipy 1.10.0, seaborn 0.12.2, pandas 1.5.3, tifffile 2021.7.2, scikit-image 0.20.0.dev0, numpy 1.23.5, matplotlib 3.7.0, dipy 1.7.0<br>The code for fusing the data from the microscope and data analyses is available on GitHub: https://github.com/fmi-basel/gliberal-lightsheet-2023<br>For data visualization ImageJ 2.9.0 and Paraview 5.10.1 was used.<br>For cell tracking Mastodon v1.0.0-beta 26 was used (https://github.com/mastodon-sc/mastodon, v1.0.0-beta-26).<br>For cell segmentation Cellpose 2.0 v2.2 was used (https://github.com/MouseLand/cellpose).

For manuscripts utilizing custom algorithms or software that are central to the research but not yet described in published literature, software must be made available to editors and reviewers. We strongly encourage code deposition in a community repository (e.g. GitHub). See the Nature Portfolio guidelines for submitting code & software for further information.

Data

Policy information about availability of data

All manuscripts must include a data availability statement. This statement should provide the following information, where applicable:

- Accession codes, unique identifiers, or web links for publicly available datasets
- A description of any restrictions on data availability
- For clinical datasets or third party data, please ensure that the statement adheres to our policy

Source data supporting the findings of this study will be made available by the corresponding authors on request.

Human research participants

Policy information about studies involving human research participants and Sex and Gender in Research.

Reporting on sex and gender	N/A
Population characteristics	N/A
Recruitment	N/A
Ethics oversight	N/A

Note that full information on the approval of the study protocol must also be provided in the manuscript.

Field-specific reporting

Please select the one below that is the best fit for your research. If you are not sure, read the appropriate sections before making your selection.

☒ Life sciences ☐ Behavioural & social sciences ☐ Ecological, evolutionary & environmental sciences

For a reference copy of the document with all sections, see nature.com/documents/nr-reporting-summary-flat.pdf

Life sciences study design

All studies must disclose on these points even when the disclosure is negative.

Sample size	No sample size calculation was performed. In general sample sizes were kept as big as practically possible with the described microscopy setup.
Data exclusions	For the analysis of cell shapes based on segmentations, false segmentations were excluded based on cell volume and values for major and minor axis. For showcasing the functionality of the presented microscope, representative specimens are shown in figures and videos.
Replication	Experiments were repeated multiple times and only results consitent between repetitions are presented in the manuscript. Where applicable, multiple structures and/or cells were used for analysis and quantification.
Randomization	No randomization was applied to select the presented data of intestinal organoids, Hydra, hepatic organoids, human colon cancer organoids, or parotid salivary gland orgnoids. Representative organoids were chosen. For cell tracking in intestinal organoids, an organoid with high morphological and cell type compositional complexity was selected. Comparative EMT related quantifications were performed on gatruloids allocated to experimental groups based on experimental time points. In general, sample randomization was not necessary to showcase the features of the light sheet microscope described in this manuscript.
Blinding	The same investigators performed data collection and analysis. Therefore no blinding was performed.

Reporting for specific materials, systems and methods

We require information from authors about some types of materials, experimental systems and methods used in many studies. Here, indicate whether each material, system or method listed is relevant to your study. If you are not sure if a list item applies to your research, read the appropriate section before selecting a response.

Materials & experimental systems

n/a	Involved in the study
☐	☒ Antibodies
☐	☒ Eukaryotic cell lines
☒	☐ Palaeontology and archaeology
☐	☒ Animals and other organisms
☒	☐ Clinical data
☒	☐ Dual use research of concern

Methods

n/a	Involved in the study
☒	☐ ChIP-seq
☒	☐ Flow cytometry
☒	☐ MRI-based neuroimaging

Antibodies

Antibodies used

The following primary antibodies were used in this study: Sheep anti Dll1 (Catalog no. AF3970, RnD Systems) and rabbit anti Lysozyme (Catalog no. A0099, Dako). Donkey anti rabbit Fab fragments conjugated to Alexa 647 and donkey anti goat Fab fragments conjugated to Alexa 488 fluorophores were used as secondary agents (Catalog no. 705-607-003 and 711-547-003, Jackson Immuno Research).

Validation

Primary antibodies were validated in a previous study: Serra, D., Mayr, U., Boni, A. et al. Self-organization and symmetry breaking in intestinal organoid development. Nature 569, 66–72 (2019). https://doi.org/10.1038/s41586-019-1146-y.
Fab fragments were validated using non primary stained samples (negaive control) and single stained samples (only one staining per sample). Further, the resulting stainigs were compared to controls using conventional secondary antibodies.

Eukaryotic cell lines

Policy information about cell lines and Sex and Gender in Research

Cell line source(s)

Human: Female patient-derived organoids identified by the HUB code P-19bT CRC organoids are cataloged at www.huborganoids.nl. Organoids were generated using a transposase-based integration method (movieSTAR: Tol2 insulator8xSTAR-min.pLGR5-sTomato-NLS-pA-PGK-H2BmNeonGreen-2A-Puro).

Mouse: both male and female mice were used to generate organoids. For "FUCCI experiments" organoids were generated from B6/N x R26 Fucci2 (Tg/+) intestines. Organoids were subsequently infected with pGK Dest H2B-miRFP670 (Catalog no. 90237, Addgene. For the remaining organoid based experiments heterzygotic R26-mG/H2B-mCherry mice were used. These mice originated from crosses of R26-mG (C57BL/6J, Muzumdar, M.D., Tasic, B., Miyamichi, K., Li, L. and Luo, L. (2007), A global double-fluorescent Cre reporter mouse. Genesis, 45: 593-605. https://doi.org/10.1002/dvg.20335) and R26-H2B-mCherry (Abe, T., Kiyonari, H., Shioi, G., Inoue, K.-I., Nakao, K., Aizawa, S., and Fujimori, T. (2011). Establishment of conditional reporter mouse lines at ROSA26 locus for live cell imaging. Genesis 49, 579–590).

mESC lines for gastruloid culture: E14 (male) and CGR8 (male) cell lines are of 129 background and were provided by the laboratory of Matthias Lutolf (Institute of Human Biology, Basel).

Authentication

Cell lines used in this study were not authenticated.

Mycoplasma contamination

Cell and organoid lines were routinely tested for mycoplasma contamination. No mycoplasma contaminated material was used in this study.

Commonly misidentified lines
(See ICLAC register)

No commonly misidentified cell lines were used for this study.

Animals and other research organisms

Policy information about studies involving animals; ARRIVE guidelines recommended for reporting animal research, and Sex and Gender in Research

Laboratory animals

Mouse:
For mG/H2B-mCherry organoids heterzygotic R26-mG/H2B-mCherry mice were used. These mice originated from crosses of R26-mG (C57BL/6J, Muzumdar, M.D., Tasic, B., Miyamichi, K., Li, L. and Luo, L. (2007), A global double-fluorescent Cre reporter mouse. Genesis, 45: 593-605. https://doi.org/10.1002/dvg.20335) and R26-H2B-mCherry (Abe, T., Kiyonari, H., Shioi, G., Inoue, K.-I., Nakao, K., Aizawa, S., and Fujimori, T. (2011). Establishment of conditional reporter mouse lines at ROSA26 locus for live cell imaging. Genesis 49, 579–590). Regarding husbandry, all mice have a 12/12 hours day/night cycle. Medium temperature is 22°C and relative humidity is at 50%. Male and female mice with an age between 5 and 7 weeks were used.
In all other cases already established organoid lines were used.

Hydra:
This study used regenerating Hydra vulgaris (ecto[β-act::RFP]/endo[β-act::GFP] “Reverse Watermelon”). Tissue pieces of adult Hydra were used to monitor Hydra regeneration and the formation of new intact individuals. Budding stage Hydra (more than two weeks since detachment) were used.

Wild animals

This study did not involve wild animals.

Reporting on sex	Sex based information was not collected in this study. Hydra used in this study were propagated asexually.
Field-collected samples	This study did not involve samples collected from the field.
Ethics oversight	Approved by Basel Cantonal Veterinary Authorities and conducted in accordance with the Guide for Care and Use of Laboratory Animals.

Note that full information on the approval of the study protocol must also be provided in the manuscript.
